# Effects of global change drivers on the expression of pathogenicity and stress genes in dryland soil fungi

**DOI:** 10.1128/msphere.00658-24

**Published:** 2024-10-30

**Authors:** Adriana L. Romero-Olivares, Andrea Lopez, Jovani Catalan-Dibene, Scott Ferrenberg, Samuel E. Jordan, Brooke Osborne

**Affiliations:** 1 Department of Biology, New Mexico State University, Las Cruces, New Mexico, USA; 2 Department of Ecosystem and Conservation Sciences, University of Montana, Missoula, Montana, USA; 3 Arizona State University, School of Life Sciences, Tempe, Arizona, USA; 4 Department of Environment and Society, Utah State University, Moab, Utah, USA; University of Wisconsin-Madison, Madison, Wisconsin, USA

**Keywords:** fungi, pathogenicity, stress, global change, physical disturbance, drought, dryland

## Abstract

The impacts of global climate change on dryland fungi have been understudied even though fungi are extremely sensitive to changes in the environment. Considering that many fungi are pathogens of plants and animals, including humans, their responses to anthropogenic change could have important implications for public health and food security. In this study, we investigated the potential physiological responses (i.e., metatranscriptomics) of pathogenicity and stress in dryland fungi exposed to global change drivers, drought, and the physical disturbance associated with land use. Specifically, we wanted to assess if there was an increase in the transcription of genes associated to pathogenicity and stress in response to global change drivers. In addition, we wanted to investigate which pathogenicity and stress genes were consistently differentially expressed under the different global change conditions across the heterogeneous landscape (i.e., microsite) of the Chihuahuan desert. We observed increased transcription of pathogenicity and stress genes, with specific genes being most upregulated in response to global change drivers. Additionally, climatic conditions linked to different microsites, such as those found under patches of vegetation, may play a significant role. We provide evidence supporting the idea that environmental stress caused by global change could contribute to an increase of pathogenicity as global climate changes. Specifically, increases in the transcription of stress and virulence genes, coupled with variations in gene expression, could lead to the onset of pathogenicity. Our work underscores the importance of studying dryland fungi exposed to global climate change and increases in existing fungal pathogens, as well as the emergence of new fungal pathogens, and consequences to public health and food security.

**IMPORTANCE:**

The effects of global climate change on dryland fungi and consequences to our society have been understudied despite evidence showing that pathogenic fungi increase in abundance under global climate change. Moreover, there is a growing concern that global climate change will contribute to the emergence of new fungal pathogens. Yet, we do not understand what mechanisms might be driving this increase in virulence and the onset of pathogenicity. In this study, we investigate how fungi respond to global change drivers, physical disturbance, and drought, in a dryland ecosystem in terms of pathogenicity and stress. We find that indeed, under global change drivers, there is an increase in the transcription and expression of genes associated to pathogenicity and stress, but that microclimatic conditions matter. Our study shows the importance of investigating dryland fungi exposed to global climate change and impacts on our society, which may include threats to public health and food security.

## INTRODUCTION

The Intergovernmental Panel on Climate Change has emphasized that the collision of global change drivers, such as physical disturbance (referred simply as disturbance here onward) and drought, in the next two decades will often breach tolerance thresholds for biological systems, with repercussions for public health (1). For example, as climate change intensifies droughts, soil dries out, which facilitates soil dispersion through wind force, consequently increasing land erosion, which is a type of disturbance (2, 3). One potentially critical but understudied impact of global change is its effect on the physiology of soil fungi (4). Many soil fungi are causal agents of infectious diseases of high prevalence and public health impact (5, 6); in fact, the direct medical cost associated with fungal diseases in the USA alone is more than $7.2 billion (6). Moreover, fungal diseases impact food security (7, 8); the cost associated with crop losses by fungal diseases is $100–$200 billion every year (9). Because global climate change is happening faster than anticipated (1), it is especially important to investigate soil fungal responses to global change drivers, as fungi are extremely sensitive to changes in the environment, and their responses could have important implications for public health and food security (4).

Fungal responses under global change drivers have been studied mostly from a community-based perspective, that is, assessments of changes in the fungal community in terms of the relative abundance of different taxonomical groups and/or functional groups (10–16). Although responses vary by global change driver and ecosystem type, a consistent finding has been the increase of pathogenic fungal taxa and/or functional groups (17, 18). Large-scale research supports this observation as fungal pathogenic outbreaks have been increasingly documented in the last decade and are predicted to continue to increase (19–22). The mechanisms behind the increases of pathogenic fungi under global change drivers remain unknown.

An interesting hypothesis regarding the rise of fungal pathogens due to global climate change suggests that increased stress resilience in fungi may enhance their virulence, leading to a higher prevalence of pathogenicity under global climate change (5). In fact, the novel fungal pathogen *Candida auris*, which was first identified in 2009 from an ear infection (23), is thought to have emerged due to exposure to chronic stress in its natural environment imposed by global climate change (24–26). Prior to becoming pathogenic, *C. auris* was likely a saprotrophic fungus. These ideas are supported by the fact that its closest phylogenetic relatives have been isolated from aquatic environments (26), and *C. auris* can tolerate high-stress environments such as hypersalinity and higher temperatures compared to other pathogenic *Candida* species (27, 28).

Aside from human health, food systems are also at risk of fungal pathogens under global climate change. Agroecosystems, especially those growing global commodity crops, such as banana, coffee, tomato, cotton, etc., are threatened by emerging fungal pathogens. For example, *Fusarium oxysporum* f. sp. *cubense*, causal agent of banana wilt, was responsible for the eradication of the Gros Michel banana in the 1960s (8). A new banana cultivar, Cavendish, is now popular, but it is currently threatened because of a recently emerged variant of *F. oxysporum* f. sp. *cubense*, also known as tropical race 4 (TR4). The cause of the emergence of this variant is unknown (although likely due to management practices), and its spread may be exacerbated by global climate change (29). The emergence of plant pathogens and their impact on food security remain a subject of ongoing research. For instance, the devastation of banana crops by TR4 forced Colombia, a leading banana exporter, to declare a state of emergency (30). To fully understand the threat, it is essential to investigate how pathogens respond to global change drivers and their evolutionary capacity to withstand environmental stress. Regardless, the resilience of agricultural systems and the subsequent impacts on food security will be challenged by climate change, as pathogens are likely to follow hosts as they disperse globally and evolve to overcome environmental stresses (8).

Regardless of the type of fungal pathogen (human or plant), the connection between the ability to withstand stress and increases in virulence which can result in the onset of pathogenicity is clear; the inside of a host is often a stressful environment. Depending on the host, there might be limited carbon sources, elevated temperature, and an active immune system. Pathogenic fungi, both obligate and facultative, have evolved strategies to withstand these conditions and facilitate host invasion. For example, to establish infection, *Candida albicans* activates a stress response pathway that results in changes in the structure, biophysical properties, and architecture of the cell wall (31). Moreover, enzymes, such as multicopper oxidases and metalloproteases, are produced by many fungi, and their role is very broad and includes participating in the degradation of carbon, as well as functioning as a virulence factor (32, 33).

Although the connections between virulence and stress tolerance in fungi seem to be clear, these studies have been done mostly in model species under controlled laboratory conditions (32, 34–38). To our knowledge, these connections have not been investigated in fungal communities in natural soil environments experiencing global change. Therefore, in this study, we answer the following questions: (i) is there an increase in the transcription of genes associated to pathogenicity and stress in response to global change drivers? (ii) Is the expression of pathogenicity and stress genes higher under global change drivers? (iii) Which pathogenicity and stress genes are consistently differentially expressed under global change drivers? Finally, (iv) how does the gene expression of the fungal community respond to individual and overlapping global change drivers?

**Fig 1:**
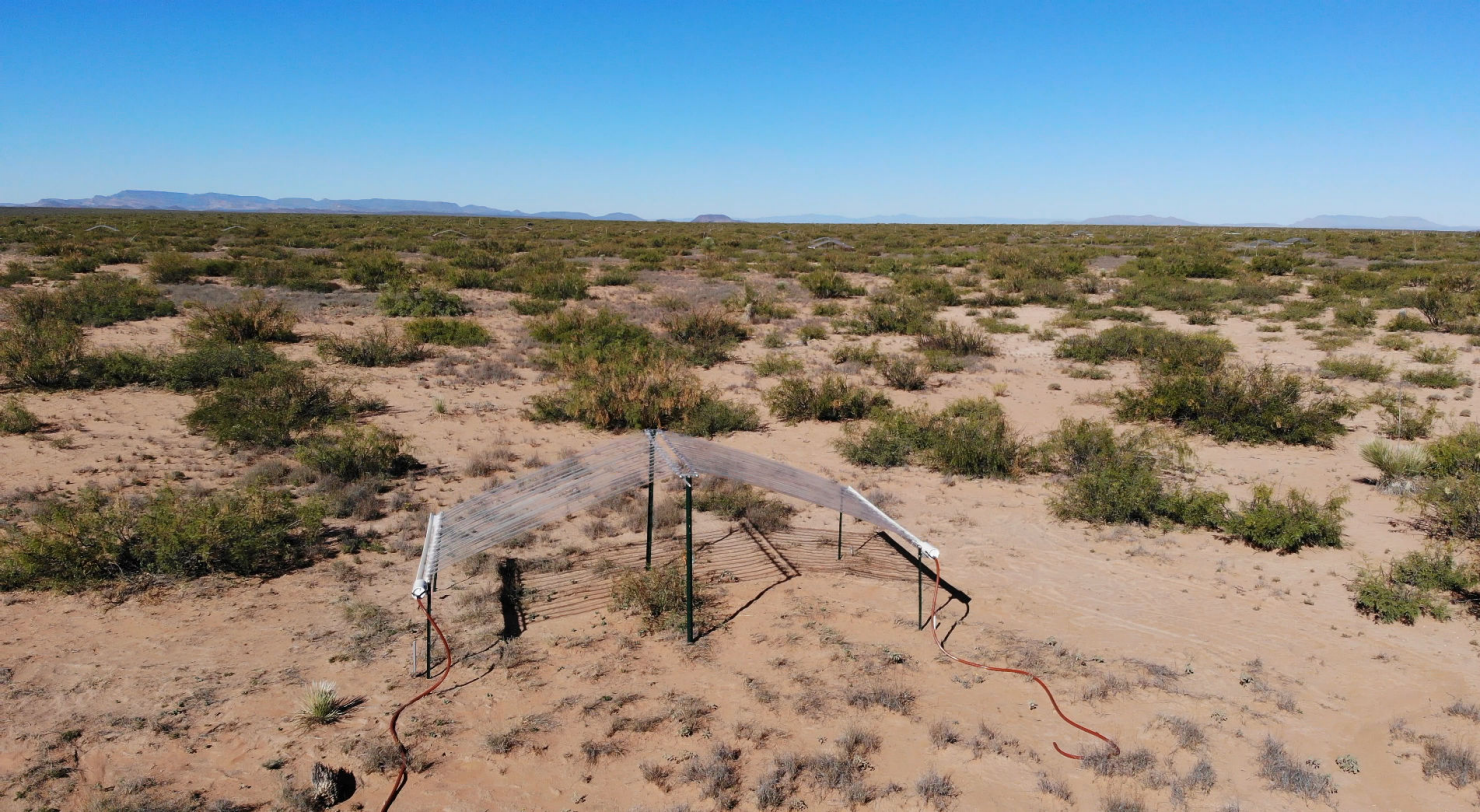
View of the heterogeneous landscape of shrubs separated by interspace areas of open soil in our field experiment in the northern extent of the Chihuahuan Desert at the Jornada Basin LTER (Photo credit: Scott Ferrenberg).

To address these questions, we conducted research at the Jornada Basin LTER (long-term ecological research) in the northern extent of the Chihuahuan Desert (i.e., dryland ecosystem) in a manipulative field experiment using disturbance and drought as global change drivers imposed in a full-factorial design. This site has been experiencing land degradation for the last century due to global climate change and other anthropogenic activities (39, 40). These impacts have helped create a heterogeneous landscape (39, 41, 42) with patches of vegetation separated by interspace areas of open soil (Fig. 1). This “patchiness” makes the landscape susceptible to further land degradation, such as erosion, that can contribute to additional disturbance of the desert floor due to dust storms (43). But this heterogeneous landscape also offers the opportunity to study the responses of the fungal community to global change drivers under different microclimatic conditions, such as the presence or absence of vegetation. Altogether, this will allow us to better understand how these conditions may influence how the fungal community responds to global climate change and identify potential implications for public health and food security.

## MATERIALS AND METHODS

Our study was located at the Jornada Basin LTER site (32° 30′ N, 106° 47′ W, 1,188 m. a. s. l.), in the drylands of the northern extent of the Chihuahuan Desert in southern New Mexico, USA. This site has a mean annual precipitation of 230 mm with a marked monsoon season from July to October. Average maximum temperature is 36°C usually occurring in early summer, while the average minimum temperature is 13°C usually occurring in early winter. The dominant vegetation consists of shrubs such as honey mesquite (*Prosopis glandulosa*), creosote bush (*Larrea tridentata*), and tarbush (*Flourensia cernua*), which have been increasing in abundance for the last few decades (i.e., shrub encroachment) (44, 45).

In a honey mesquite-dominated area of approximately 600 × 400 m, 40 field plots measuring 2.5 × 5 m were installed in 2019 and randomly assigned to four experimental treatments. We selected the plot size to capture the ecological dynamics of the dominant shrub species in this study as well as the interspace immediately surrounding the shrubs. Spacing among plots was irregular since dryland vegetation is heterogeneous in space, and plots were centered on individual shrubs so that vegetation was similar across all plots prior to treatment; selected shrubs were typical of each site in size, number of stems, and canopy vigor.

Of these 40 plots, 10 were physically disturbed at the start of the experiment with multiple passes with a spiked drum aerator pulled with an all-terrain vehicle that damaged plant and soil communities. The purpose of the disturbance treatment was to impose soil surface disturbances such as those caused by anthropogenic land use, like vehicle traffic. Ten plots were droughted using rainout shelters that impose a 70% reduction of incoming precipitation (46), which, according to the long-term data at the Jornada, represents an extreme, 1-in-100-year drought event for the area. Ten plots were both disturbed and droughted (D × D), and 10 plots were left untouched to serve as control.

We collected soil samples from each plot 2 years after the onset of the global change experiment. In each plot, we collected soil samples from two different microsites, (i) under vegetation, which are areas below plant patches where there is significant accumulation of organic matter and nutrients, also known as “resource islands” and which are major drivers of dryland ecosystem functioning (45, 47–49); (ii) from interspaces which are adjacent open areas of soil (i.e., open soil with or without biological soil crusts; total of 80 soil samples). We collected approximately 1 g of soil from the top 5 cm where microbial activity is often greatest and immediately soaked it in 5 mL of LifeGuard Soil Preservation Solution (Qiagen Group), where RNAse activity is prevented, and RNA microbial community profiles are maintained and stabilized (50). Samples were kept in a cooler for a few hours and then transferred to a −80°C freezer upon arrival at the lab and processed within 2 months of collection.

We thawed samples on ice and centrifuged at 2,500 × *g* for 5 minutes to remove the LifeGuard Soil Preservation Solution and proceeded to extract RNA using the RNA PowerSoil following the manufacturer’s instructions with some modifications (51). We cleaned and concentrated samples using the RNA clean and concentrator-25 kit (Zymo Research Corporation) and treated the samples with Turbo-DNA free kit (Life Technologies). We checked RNA for quality via electrophoresis, and those samples with enough RNA concentration and of good quality were shipped to the Center for Genomics and Bioinformatics at Indiana State University (Bloomington, IN, USA) for sequencing. Here, polyA-selected mRNA libraries were prepared by Illumina TruSeq Stranded mRNA Library Preparation Kit protocol and analyzed by Agilent 4200 TapeStation. The libraries were pooled and loaded on a NextSeq 500/550 High Output (v 2.5; 300 cycle) flow cell to generate paired-end reads which were then demultiplexed using Illumina’s bcl2fastq (v 2.20.0).

We selected two metatranscriptomes [minimum number of metatranscriptomes needed to be able to successfully conduct differential expression analyses (52)] for control and each treatment (total of 16) based on comparable number of reads (Table S1) and analyzed following Romero-Olivares and collaborators (53). Briefly, we removed adapters with Trimmomatic (v 0.39) using ILLUMINA TruSeq3-PE adapters with sliding window 4:15 and dropping reads below 25 bases long (54). We checked the quality of trimmed samples with FastQC (v 0.11.9) (55). We removed 5, 5.8, 16, 18, and 23 s rRNA sequences with sortmeRNA (56) and the SILVA database (57). We assembled a *de novo* reference metatranscriptome with Trinity (v 2.13.2) (58) and used bowtie2 (v 2.4.5) to map reads (59) and samtools (v 1.15) for sorting and indexing (60). We annotated our metatranscriptome using the Pfam (v 36.0) protein family database which is used for classifying protein sequences into families and domains (61). We used Transdecoder (v 5.5.0) (62) to find coding regions, Trinotate for annotations (v 3.2.2) (63), and hmmer (v 3.3.2) (64) to search for sequence homologs. We ran this pipeline two times based on microsite (i.e., interspace samples and under vegetation samples) due to computational demands associated to the size of our files which, in most cases, exceeded one terabyte. Once we had an annotated metatranscriptome for each microsite, we used Salmon (v 1.10.2) (65) to quantify transcripts and create a gene-level count matrix.

To filter transcripts of genes associated with proteins involved in pathogenicity and stress, we conducted a literature review to identify proteins that are known for playing a role in the pathogenicity of microorganisms and/or stress response. For the former, we selected transcripts that codify for genes associated to adhesins (66), agglutinins (67–69), flocculins (70–72), melanin biosynthesis (73, 74), metalloproteins (34, 75–77), toxin (78, 79), and multicopper oxidase (32, 35, 36) (Table 1; Tables S2 and S3). For the latter (i.e., stress response), we selected transcripts that codify for genes associated to β−1,3 glucan synthase (80–82), heat shock protein (HSP) (82, 83), melanin biosynthesis (84, 85), RNA helicase (82, 86, 87), and trehalose (87, 88) (Table 1; Tables S4 and S5). In addition, we conducted an extensive analysis of our Pfam outputs using the Pfam/InterPro database (89) and read the description of each protein. We selected transcripts that codify proteins with the term “pathogenicity,” “virulence,” or “stress” in either its name and/or description (i.e., target name and description of target, Tables S2 to S5). We used count matrices for the transcripts of interest, that is, pathogenicity and stress, and ran DESeq2 package (v 1.42.0) within Bioconductor (v 3.18) (52) in R (90) to conduct differential analysis of transcript count data. For plots and statistical analyses, we used the output of DESeq2 [i.e., differentially expressed gene (DEG) data] which provides log2fold change data that show the increased expression of a specific gene in control compared to treatment by a multiplicative factor of 2. We used the output of Salmon (i.e., gene level count matrix) which provides the total number of transcripts for specific genes.

**TABLE 1:** Proteins included in this study known for playing a role in the pathogenicity and stress response of microorganisms

Protein name	Pathogenicity	Stress response
Adhesins	Used by pathogens to establish infection by facilitating interactions with the external environment, including the host (72, 91).	
Agglutinins	Participate in adhesion of the cell wall to host and to environmental abiotic surfaces (67).	
Flocculins	A type of adhesin found in the cell wall; it mediates cell-to-cell aggregation and is crucial for biofilm formation during infection (37, 70).	
Melanin biosynthesis	Cell wall polymer that can act as a virulence factor and increases resistance of cells to the immune system (e.g., resistant to phagocytosis) (73).	Cell wall polymer that ameliorates environmental stress such as UV radiation, osmotic stress, and high temperature (92).
Metalloproteins	Essential for pathogens as a virulence factor to acquire and control metal utilization during infection to survive in their hosts (75, 93).	
Toxins	Virulence factors that alters the host cell functions to facilitate infection (79, 94).	
Multicopper oxidases	A copper-containing protein that acts as virulence factor by helping evade the toxic high-metal environment generated by the host immune system (35, 95).	
β−1,3 glucan synthase		A cell wall carbohydrate that provides strength, resistance, and integrity to the cell (87).
HSP		Have a crucial role in protein folding and stability, as well as in homeostasis under stressful biotic and abiotic conditions (83, 96).
RNA helicase		Molecular motors that rearrange RNA secondary structure and are associated with response to temperature stress (87, 97).
Trehalose		A sugar that acts as protectant against abiotic stress by stabilizing proteins from desiccation (87, 98).

We conducted nested one-way ANOVAs with microsite nested within treatment as independent variable and transcript counts (with log-transformed data) or differential expression (i.e., log2fold change) as dependent variable and conducted Tukey honest significant differences as post hoc test. In all cases, we used *P* values equal or smaller to 0.05 as significant. The full pipeline, raw data on pathogenicity and stress DEGs, gene level count matrix, as well as statistical scripts were deposited at https://github.com/adriluromero/adriluromero-Jornada_DxD_RNAseq (99).

## RESULTS

### Is there an increase in the transcription of genes associated to pathogenicity and stress in response to global change drivers?

**Fig 2:**
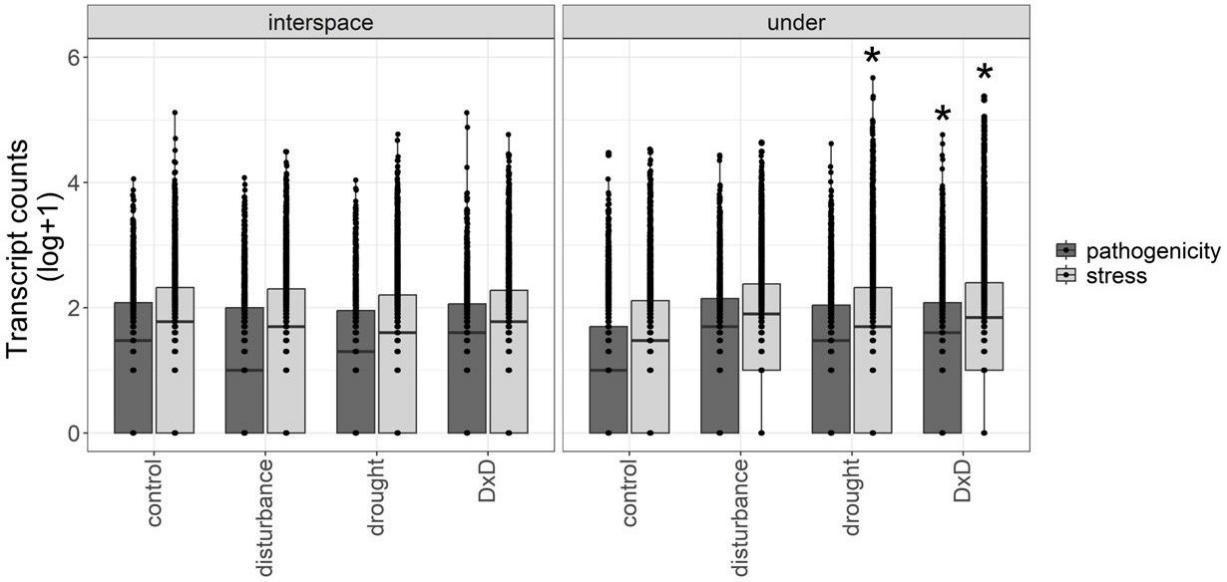
Transcript counts for pathogenicity and stress genes in control and treatments from the different microsites [interspace among plants (i.e., interspace) or under the canopy of the shrub, honey mesquite (i.e., under)] at the Jornada LTER global change experiment. Experimental treatments were control, physical disturbance, drought, and physical disturbance plus drought (D × D). Box and whisker plots show the distribution of the data, the mean, and lower and upper quartiles. Each point represents the transcript count of a specific gene. Counts are based on the sum of two metatranscriptomes for control and treatment plots (*n* = 2). Asterisks denote significance at *P* ≤ 0.05 between control and treatments by microsite for pathogenicity and stress genes.

We found that there is a higher number of pathogenicity and stress transcripts in response to global change drivers, but only under vegetation and only for specific treatments. For pathogenicity genes, the fungal community in under vegetation and interspaces had comparable number of transcripts between control and treatments (treatment:microsite F_4,23788_ = 1.17, *P* = 0.318; Fig. 2). However, a post hoc test revealed significantly higher pathogenicity transcript counts in D × D compared to control (*P* = 0.011, Fig. 2) in under vegetation. For stress genes, there were significant differences in the number of transcript counts between control and treatments in the different microsites (treatment:microsite F_4,42952_ = 12.31, *P* < 0.001; Fig. 2). Post hoc test showed that there were significantly higher number of stress transcripts under drought (*P* ≤ 0.001) and D × D (*P* ≤ 0.001) under vegetation compared to control.

### Is the expression of pathogenicity and stress genes higher under global change drivers?

**Fig 3:**
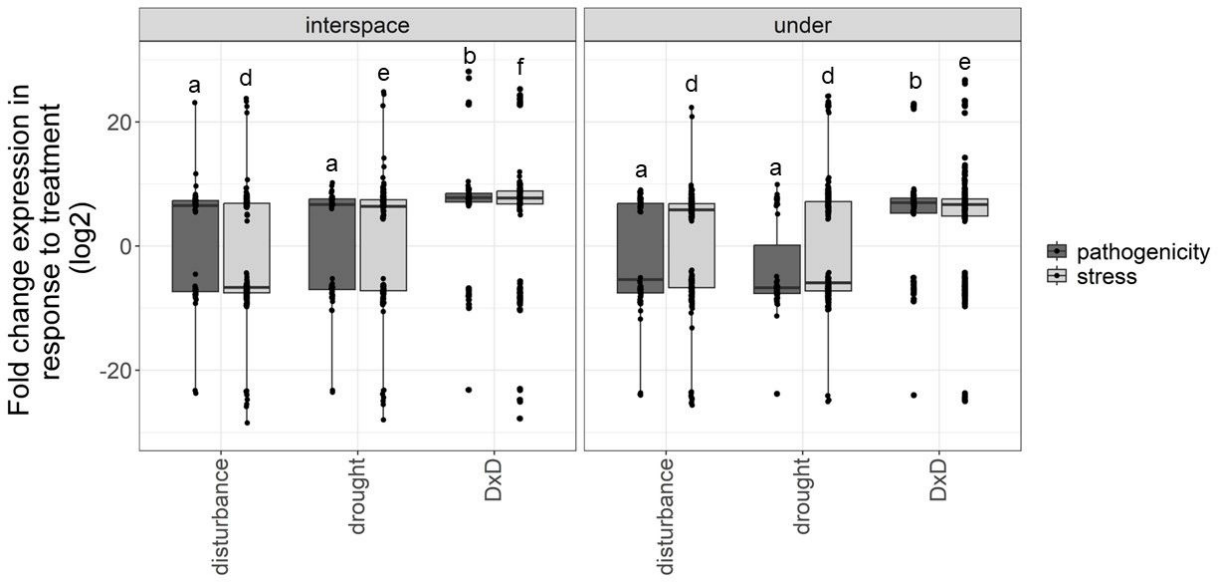
DEGs for pathogenicity and stress genes in treatments in comparison to control from the different microsites [interspace among plants (i.e., interspace) or under the canopy of the shrub, honey mesquite (i.e., under)] at the Jornada LTER global change experiment. Experimental treatments were control, physical disturbance, drought, and physical disturbance plus drought (D × D). Box and whisker plots show the distribution of the data, the mean, and lower and upper quartiles. Each point represents the fold change expression of a gene in treatment compared to control. Differential expression is based on the data of two metatranscriptomes for control and treatment plots (*n* = 2). Different letters denote significance at *P* ≤ 0.05 by microsite for pathogenicity and stress genes.

The expression of pathogenicity and stress genes in interspace and under vegetation was high in response to global change drivers, especially under D × D compared to disturbance and drought alone (pathogenicity treatment:microsite F_3,453_ = 5.78, *P* < 0.001; stress treatment:microsite F_3,1776_ = 9.16, *P* < 0.001; Fig. 3). The expression of pathogenicity genes was comparable between disturbance and drought alone in interspace (*P* = 0.999) and under vegetation (*P* = 0.375). Similarly, the expression of stress genes was comparable between disturbance and drought alone in under vegetation (*P* = 0.611) but significantly lower in disturbance compared to drought in interspace (*P* < 0.001).

### Which pathogenicity and stress genes are consistently differentially expressed under global change drivers?

**Fig 4:**
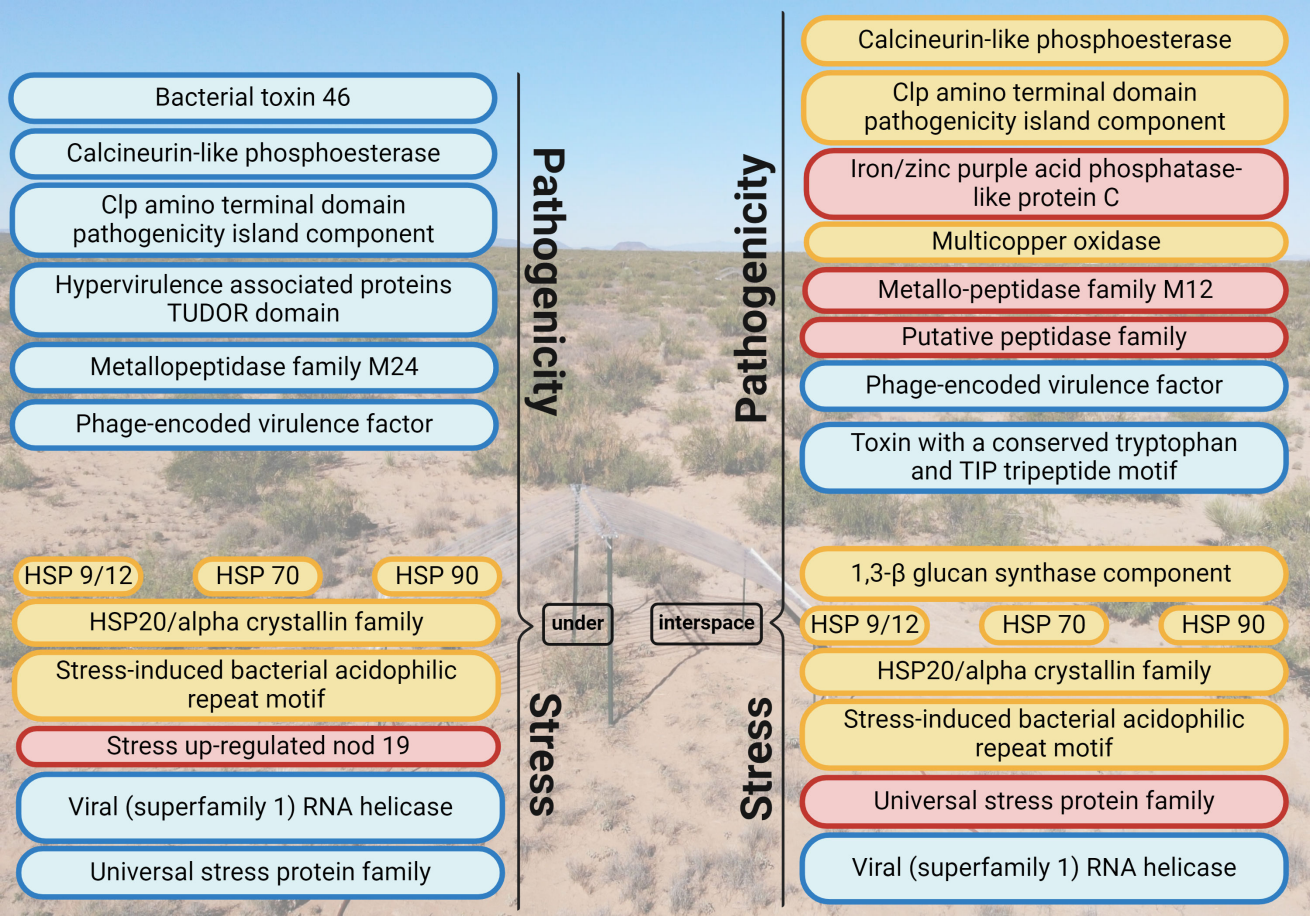
Consistently DEGs for pathogenicity and stress in response to all global change drivers in interspaces and under vegetation. Red labels highlight genes that are consistently upregulated under global change drivers, blue labels highlight genes that are consistently downregulated under global change drivers, and yellow labels are genes that are consistently, both, up- and downregulated under global change drivers. Illustration created with BioRender.com, license agreement OE274MO1 × 9.

*Calcineurin-like phosphoesterase*, *clp amino terminal domain pathogenicity island component*, and *phage-encoded virulence factor* where pathogenicity genes are consistently differentially regulated in response to global change drivers in both microsites. However, its regulation varied; *phage-encoded virulence factor* was consistently downregulated, while genes encoding *calcineurin-like phosphoesterase* and *clp amino terminal domain pathogenicity island component* were up and downregulated. Interestingly, there were no pathogenicity genes that were consistently upregulated in under vegetation in response to global change drivers (Fig. 4). Contrastingly, in interspaces, *iron-zinc purple acid phosphatase-like protein C*, *metallo-peptidase family M12*, and *putative peptidase family* were consistently upregulated under global change drivers. HSP *20/alpha crystallin family*, *HSP 9/12*, *HSP 70*, *HSP 90*, and *stress-induced bacterial acidophilic repeat motif* were stress genes that were consistently expressed under global change drivers in both microsites, although its regulation varied. *Viral (superfamily) RNA helicase* was consistently downregulated under global change drivers in both microsites. Contrastingly, *stress upregulated nod 19* and *universal stress protein family* were consistently upregulated in under vegetation and interspace, respectively (Fig. 4).

### How does the gene expression of the fungal community respond to individual and overlapping global change drivers?

**Fig 5:**
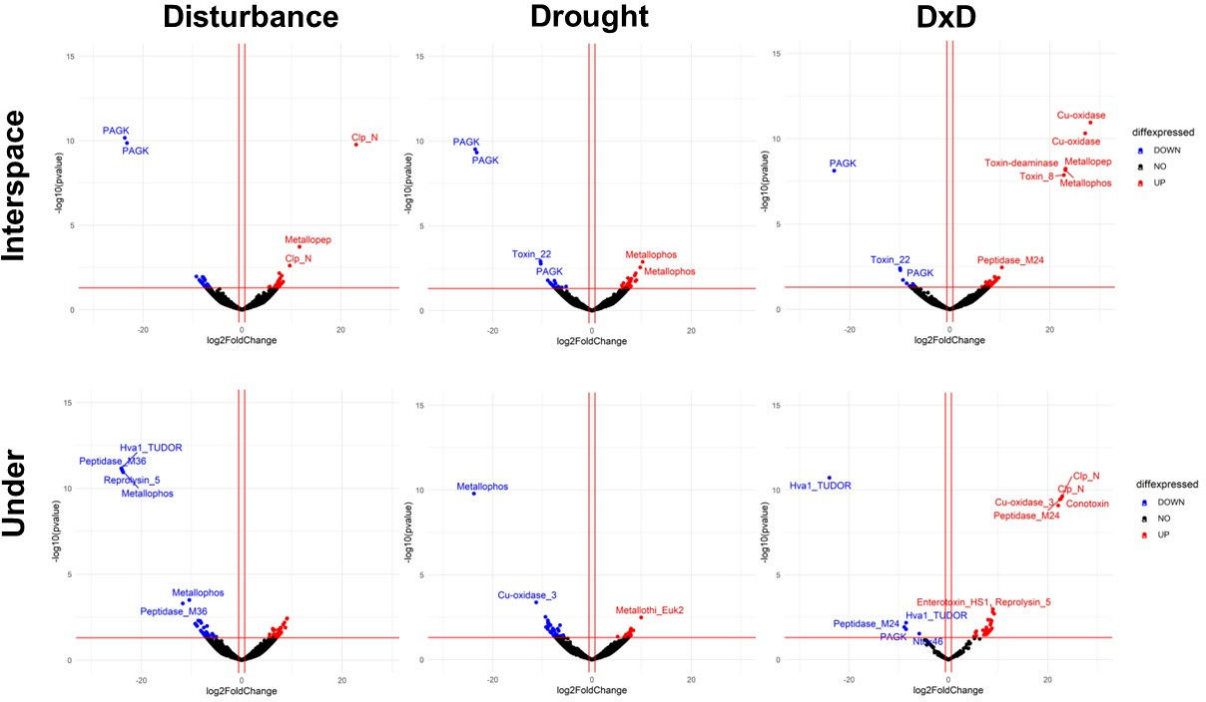
Volcano plots show significantly differentially expressed pathogenicity genes (i.e., *P* ≤ 0.05) in treatments in comparison to control from the different microsites [interspace among plants (i.e., interspace) or under the canopy of the shrub, honey mesquite (i.e., under)] at the Jornada LTER global change experiment. Experimental treatments were control, physical disturbance, drought, and physical disturbance plus drought (D × D). Blue shows significantly differentially downregulated genes, red shows significantly differentially upregulated genes, and black shows genes that were not significantly up- or downregulated. Differential expression is based on the data of two metatranscriptomes for control and treatment plots (*n* = 2).

We saw more similarities in the expression of pathogenicity genes by microsite compared to treatments (Fig. 5). Microsites, for example, responded similarly in downregulation of pathogenicity genes. In interspaces, *phage-encoded virulence factor (PAGK*) consistently showed the most downregulation across all treatments, while under vegetation, *hypervirulence-associated protein TUDOR domain* (Hva1 TUDOR) consistently exhibited the most downregulation in response to all treatments (Fig. 5). In interspaces, in disturbance alone, *clp amino terminal domain pathogenicity island component* (clp N) was the most upregulated gene, whereas in drought alone, it was *calcineurin-like phosphoesterase* (metallophosphatase). However, when disturbance and drought interacted (i.e., D × D), *multicopper oxidase* (Cu-oxidase) was the most upregulated. In the case of under vegetation, the most upregulated gene was the same in disturbance and drought alone, *metallo-beta-lactamase superfamily protein* (lactamase B). But under D × D, the highest upregulated gene was *clp amino terminal domain pathogenicity island component* (clp N; Fig. 5).

**Fig 6:**
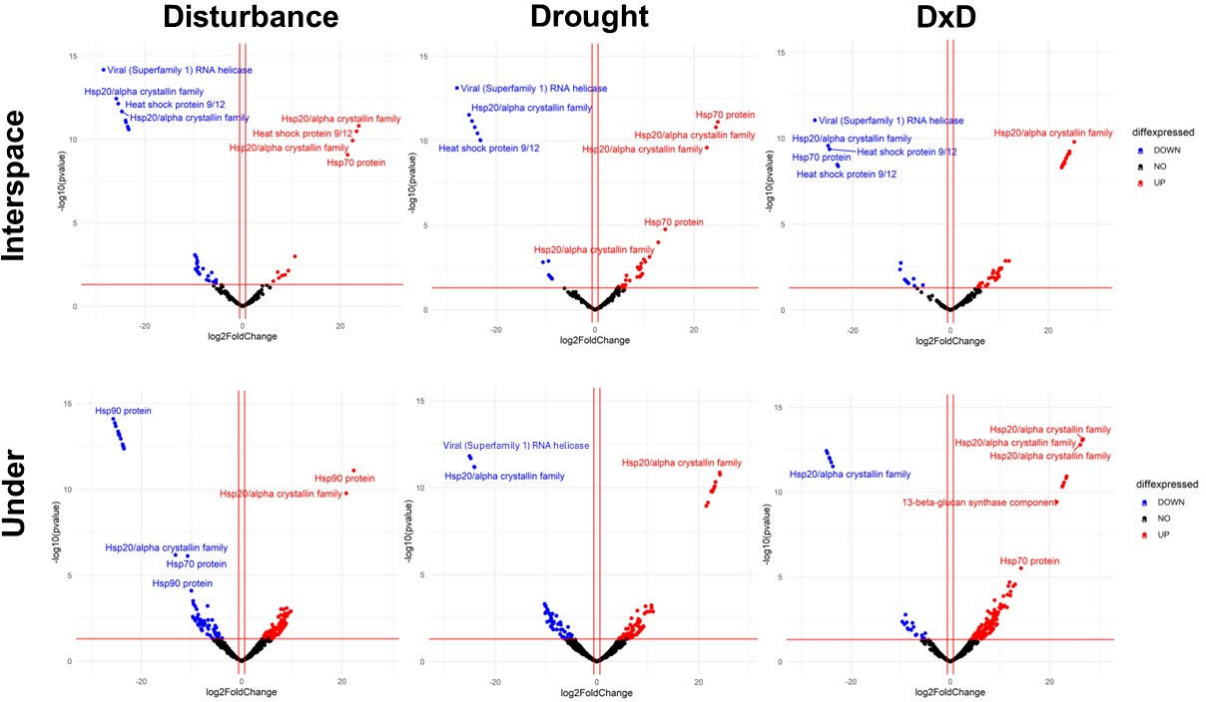
Volcano plots show significantly differentially expressed stress genes (i.e., *P* ≤ 0.05) in treatments in comparison to control from the different microsites [interspace among plants (i.e., interspace) or under the canopy of the shrub, honey mesquite (i.e., under)] at the Jornada LTER global change experiment. Experimental treatments were control, physical disturbance, drought, and physical disturbance plus drought (D × D). Blue shows significantly differentially downregulated genes, red shows significantly differentially upregulated genes, and black shows genes that were not significantly up- or downregulated. Differential expression is based on the data of two metatranscriptomes for control and treatment plots (*n* = 2).

For stress genes, we saw the same genes differentially expressed in the different microsites and treatments (Fig. 6). In other words, we did not find high variation in genes or gene expression by treatment or microsite. In interspaces, *viral* (*superfamily 1*) *RNA helicase* was the most downregulated gene across all treatments, while under vegetation, genes varied; in disturbance alone, *HSP 90* was the most downregulated, in drought alone, it was *viral* (*superfamily 1*) *RNA helicase*, and in D × D, it was *HSP 20 crystallin family* (Fig. 6). In interspaces, *HSP 20/alpha crystallin family* was the most upregulated gene in disturbance alone and D × D, whereas for drought alone, the most upregulated gene was *HSP 70*. For under vegetation, the most upregulated gene in disturbance alone was *HSP 90*, whereas drought alone and D × D had the same most upregulated gene, which was *HSP 20/alpha crystallin family* (Fig. 6).

## DISCUSSION

Transcript counts of pathogenicity and stress genes were consistent in interspaces between control and treatments (Fig. 2), suggesting that disturbance, drought, and the combination of both (i.e., D × D) did not affect the transcription of stress or pathogenicity genes of the fungal community. Contrastingly, under vegetation, we saw a significantly higher number of transcripts for both pathogenicity and stress genes in D × D compared to control (Fig. 2). Similarly, stress transcript counts for stress genes were also significantly higher under drought compared to control. Disturbance alone did not have a strong impact on the transcription of stress genes. Therefore, it is likely that the response we saw in D × D is driven mostly by the effect of drought (Fig. 2). Indeed, some fungi, such as black microcolonial fungi, are known to withstand high levels of drought as they inhabit bare rock surfaces in hot and cold deserts (100). They can withstand chronic desiccation by producing small HSPs and chaperon proteins which allow them to have a very quick response to increased water availability and for being able to function metabolically under low cellular water content (100). It is possible that fungi, in our study, were expressing HSPs in order to withstand stress, such as that imposed by our treatments under vegetation, particularly our drought treatments in drought alone and D × D. Higher transcription of pathogenicity and stress genes under D × D in under vegetation suggests that fungal access to resources, such as those found concentrated in fertile islands under vegetation, may be important in determining fungal responses to global change. For example, microsites under vegetation are probably a more competitive environment compared to interspaces and therefore, more stressful, leading to greater evolutionary selection pressures on fungal phenotypes.

Fold change expression of both pathogenicity and stress genes was significantly higher under D × D compared to disturbance and drought alone, in both interspace and under vegetation (Fig. 3). These findings provide evidence that could support the hypothesis that increased stress could lead to increases in virulence and consequently the onset of pathogenicity (5, 24). That is, under the added stress of disturbance and drought, the fungal community is expressing and regulating stress and pathogenicity genes at higher fold change compared to control and disturbance and drought alone (Fig. 3).

Previous work identified *HSP 70* and *90* as consistently upregulated in response to warming and drying in two fungal species in natural soil environments (101). These proteins are known for having a role in heat stress and pH stress (83). Our study found that these stress-related genes were consistently up- or downregulated across all treatments (Fig. 4). In contrast, the expression of pathogenicity genes varied more. Nonetheless, some genes, such as *calcineurin-like phosphoesterase*, were consistently up- or downregulated in response to nearly all treatments (Fig. 4). This protein is crucial for the virulence of fungal pathogens, facilitating key morphological changes like dimorphic transitions in animal pathogenic yeasts and appressorium formation in plant pathogens (102); in both cases, these changes are necessary for the onset of infection. The observed simultaneous up- and downregulation of some pathogenicity and stress genes suggests differential responses within the fungal community, where some members upregulate one gene, while others downregulate the same gene. This finding is significant because genes that exhibit consistent regulation in response to global change drivers may be subject to evolutionary selection pressures (103).

The regulation of HSPs varied widely under the different treatments and microsites; in some cases, the same HSP was the most down- and upregulated protein, such as in the case of *HSP 20/alpha crystallin family* in D × D soils under vegetation (Fig. 6). This gene is a conserved domain in HSPs that play an important role in many cellular processes. Therefore, the up- and downregulation of this gene, in addition to the reasons mentioned previously (i.e., differential responses within the fungal community), could also be indicating differential expression of different HSPs containing this domain. For instance, *HSP 20*, *HSP 30*a, and *HSP 20b* all have the *HSP 20/alpha crystallin family domain* gene (104). This domain is conserved across kingdoms and present in every fungal species (83). Thus, it is not unexpected to see such a broad presence of transcripts for this gene in our data set at varied degrees of regulation.

Pathogenicity DEGs varied more compared to stress genes. For pathogenicity, we saw more similarities in DEG between microsites than within treatments, whereas for stress genes, we saw more consistency on the genes that were differentially regulated in microsites and treatments (Fig. 5). This shows that the expression of pathogenicity genes is likely more specific compared to that of stress. This is expected; all fungi have pathways for stress response, and not all fungi have pathways for pathogenicity since not all fungi are obligate or facultative pathogens (105). Therefore, the DEGs we saw for pathogenicity might be associated with specific members of the community inhabiting specific treatments and microsites. For example, previous work from our group identified high heterogeneity in the taxonomical composition of the fungal community in control and treatments plots, where some unique taxa were only present under specific microclimates and in specific treatments (106).

In some cases, the genes that were the most up- or downregulated varied by treatment and microsite. In other cases, the same genes were consistently the most down- or upregulated in specific treatments and microsite. For example, the pathogenicity gene *PAGK* was the most downregulated gene in all treatments in interspaces (Fig. 5). This gene is responsible for producing exotoxins in microbes. It is possible that this gene was downregulated in interspaces because toxin production is energetically costly (94). Under harsh environmental conditions typical of interspaces (i.e., high temperatures, low nutrient availability, and the absence of a host), microbes may not be able to afford the energetic expense of toxin production. Pathogenic genes for metalloproteins were consistently regulated to the highest degree in most treatments and microsites (Fig. 5). However, the level of upregulation, in most cases, was smaller than the degree of downregulation for the most downregulated proteins (e.g., *PAGK*). Metalloproteins, such as copper, iron, and zinc-binding proteins, are important for the virulence of pathogenic fungi (75). However, an excess in the uptake of metals can lead to metal-induced cell toxicity (107). Thus, microbes require careful balance between upregulation of metalloproteins for the uptake of metals while avoiding cell toxicity. It is possible that this is one of the main reasons why the upregulation of metalloproteins was moderate in our sites (Fig. 5). The stress gene that was the most downregulated consistently under all treatments in interspaces was *Viral* (*Superfamily I*) *RNA helicase* (Fig. 6). As conditions are harsh in interspaces, as mentioned previously, it is likely that many microbes were investing resources in efficiently arranging transcripts for expression (97).

Our results, although broad and complex, offer a glimpse of the potential pathogenic and stress physiology of the fungal community under global change drivers in a dryland ecosystem. However, findings are the result of a single sampling effort conducted in early summer throughout a few hours in the morning in a mesquite-dominated site. Therefore, the transcriptomic profiles we see might be unique to that day, time, and landscape. To better understand if the transcription profiles observed in our study remain consistent over time and space, ongoing surveillance of our site and neighboring sites with diverse vegetation is essential. This should include regular soil sampling—daily, weekly, and seasonally—since studies have demonstrated that microbial communities are highly dynamic and vary with season and landscape characteristics (108–110).

Our bioinformatics pipeline relies on available databases such as Pfam within InterPro (89) and is based on Markov models which predict the best gene alignment based on multiple transcript sequence alignments (61). Because of this, the gene identities we got might, in some cases, not be fungal specific (e.g., *phage-encoded virulence factor or Neisseria toxin MafB*). These results indicate that the transcript alignment was most closely matched to available gene or genome annotations, which, in many cases, were not specific to fungi. Since we conducted polyA selection, we assume that all, or most, of our transcripts belong to Eukaryotes. However, it is possible that some non-poly A mRNA might have escaped poly A selection; consequently, some of the transcripts might not be Eukaryotic in nature. It is also possible that some of the transcripts are not of fungal origin and might belong to other Eukaryotic microorganisms such as protists; the size of our sample is very small (~1 g) and from the top 5 cm of soil, therefore unlikely that plant or animal material such as leaves, roots, or insects are present abundantly. Indeed, previous studies from our group at the Jornada have identified zero non-fungal Eukaryotic biomass in soil samples (111). Finally, the nature and function of many pathogenic and stress proteins overlap. Although, in this study, we only included melanin biosynthesis as having both a role in pathogenicity and stress (73, 92), there might be other genes that have this dual role that we did not account for. Mapping our metatranscriptomes to obligate or facultative pathogenic fungi of interest in the area such as *Coccidioides* spp., coupled with laboratory studies, would allow us to see how these fungi might be responding to different global change drivers (101). This would also allow us to see if exposure to stress increases the transcription of virulence, pathogenicity, and stress genes at the species level and provide a comprehensive understanding of the response of fungal pathogens to global climate change. Moreover, it would help us to better understand how global change drivers are impacting the stress response, virulence, and onset of pathogenicity of pathogenic fungi and determine consequences to public health and food security.

In our study, we provide evidence that global change drivers increase the number of transcripts and the expression of pathogenicity and stress genes under specific microclimatic conditions, such as those found beneath vegetation in the dryland ecosystem of the Chihuahuan Desert. In addition, we identified pathogenicity and stress genes that are consistently differentially expressed under global change drivers and which could be under evolutionary selection. Altogether, our study found evidence that supports the idea that increases in environmental stress caused by global change drivers could contribute to increases in stress tolerance and pathogenicity in the fungal community of dryland ecosystems.

## SUPPLEMENTAL MATERIAL

Table S1 msphere.00658-24-s0001.docx Number of reads in samples by treatment in interspace and under vegetation.

Table S2 msphere.00658-24-s0002.xlsx Transcripts for pathogenicity in interspace samples.

Table S3 msphere.00658-24-s0003.xlsx Transcripts for pathogenicity in under vegetation samples.

Table S4 msphere.00658-24-s0004.xlsx Transcripts for stress response in interspace samples.

Table S5 msphere.00658-24-s0005.xlsx Transcripts for stress response in under vegetation samples.

ASM does not own the copyrights to Supplemental Material that may be linked to, or accessed through, an article. The authors have granted ASM a non-exclusive, world-wide license to publish the Supplemental Material files. Please contact the corresponding author directly for reuse.
