# Supplementary material for: Effects of global change drivers on the expression of pathogenicity and stress genes in dryland soil fungi
Source: mSphere. 2024 Oct 30;9(11):e00658-24. doi: 10.1128/msphere.00658-24 (PMC11580470; doi:10.1128/msphere.00658-24)
Supplement: Table S1 — Number of reads in samples by treatment in interspace and under vegetation. [file msphere.00658-24-s0001.docx]

Supplementary Table 1. Number of reads in samples by treatment in interspace and under vegetation.

|  | Sample id | Treatment | R1  (number of reads) | R2  (number of reads) | Total  (number of reads) |
| --- | --- | --- | --- | --- | --- |
| Interspace | 301C | control | 2,255,824 | 2,224,982 | 4,480,806 |
|  | 306C | control | 3,165,953 | 3,100,558 | 6,266,511 |
|  | 327Di | disturbance | 1,510,638 | 1,485,186 | 2,995,824 |
|  | 329Di | disturbance | 1,469,963 | 1,459,452 | 2,929,415 |
|  | 314Dr | drought | 2,324,074 | 2,270,233 | 4,594,307 |
|  | 320Dr | drought | 2,186,958 | 2,147,517 | 4,334,475 |
|  | 332D×D | disturbance  and drought | 2,164,789 | 2,133,165 | 4,297,954 |
|  | 339D×D | disturbance  and drought | 2,538,537 | 2,481,777 | 5,020,314 |
| Under vegetation | 305C | control | 2214773 | 2177924 | 4,392,697 |
|  | 307C | control | 1223997 | 1230870 | 2,454,867 |
|  | 322Di | disturbance | 6156856 | 6037367 | 2,194,223 |
|  | 324Di | disturbance | 2819760 | 2783539 | 5,603,299 |
|  | 311Dr | drought | 3168866 | 3122760 | 6,291,626 |
|  | 314Dr | drought | 3697558 | 3644344 | 7,341,902 |
|  | 333D×D | disturbance  and drought | 2325452 | 2311223 | 4,636,675 |
|  | 338D×D | disturbance  and drought | 4980539 | 4793687 | 9,774,226 |
